# Supplementary figures and images for: Novel methotrexate long-acting system using ambroxol coating and hydroxypropyl methylcellulose encapsulation for preferential and enhanced lung cancer efficiency
Source: PLoS One. 2025 Jan 16;20(1):e0314941. doi: 10.1371/journal.pone.0314941 (PMC11737749; doi:10.1371/journal.pone.0314941)

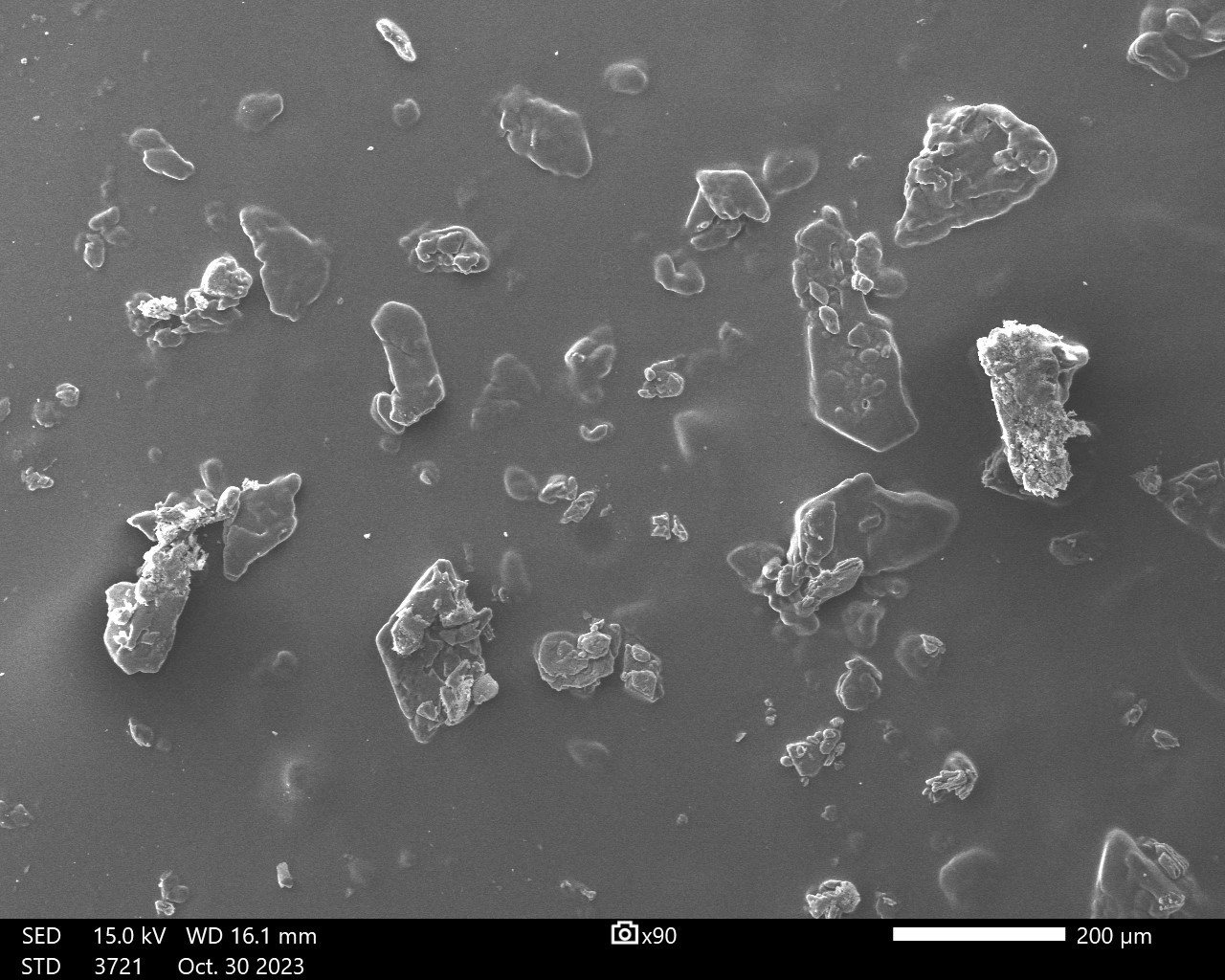

Supplement: S1 Fig — (JPG) [file pone.0314941.s001.jpg]

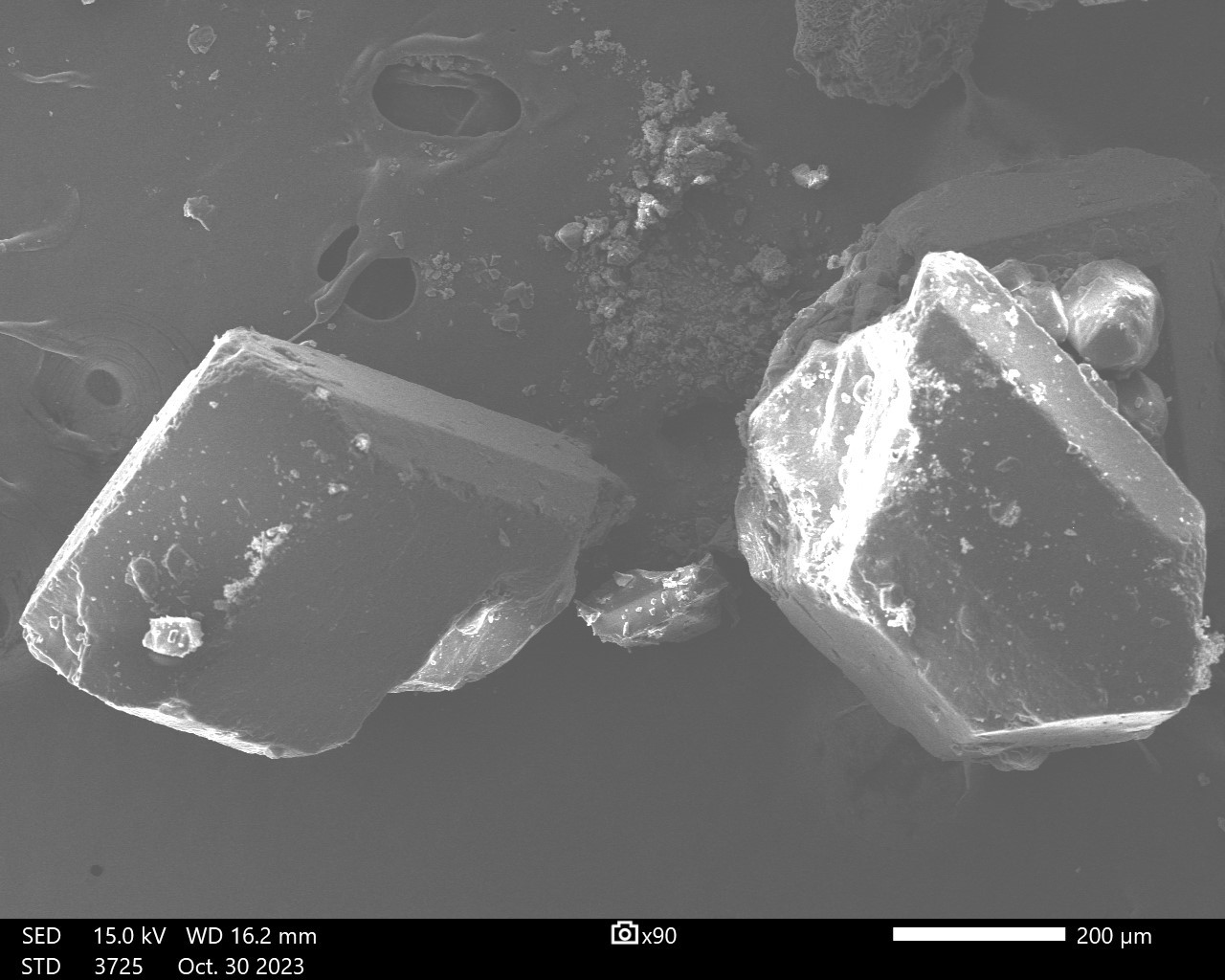

Supplement: S2 Fig — (JPG) [file pone.0314941.s002.jpg]

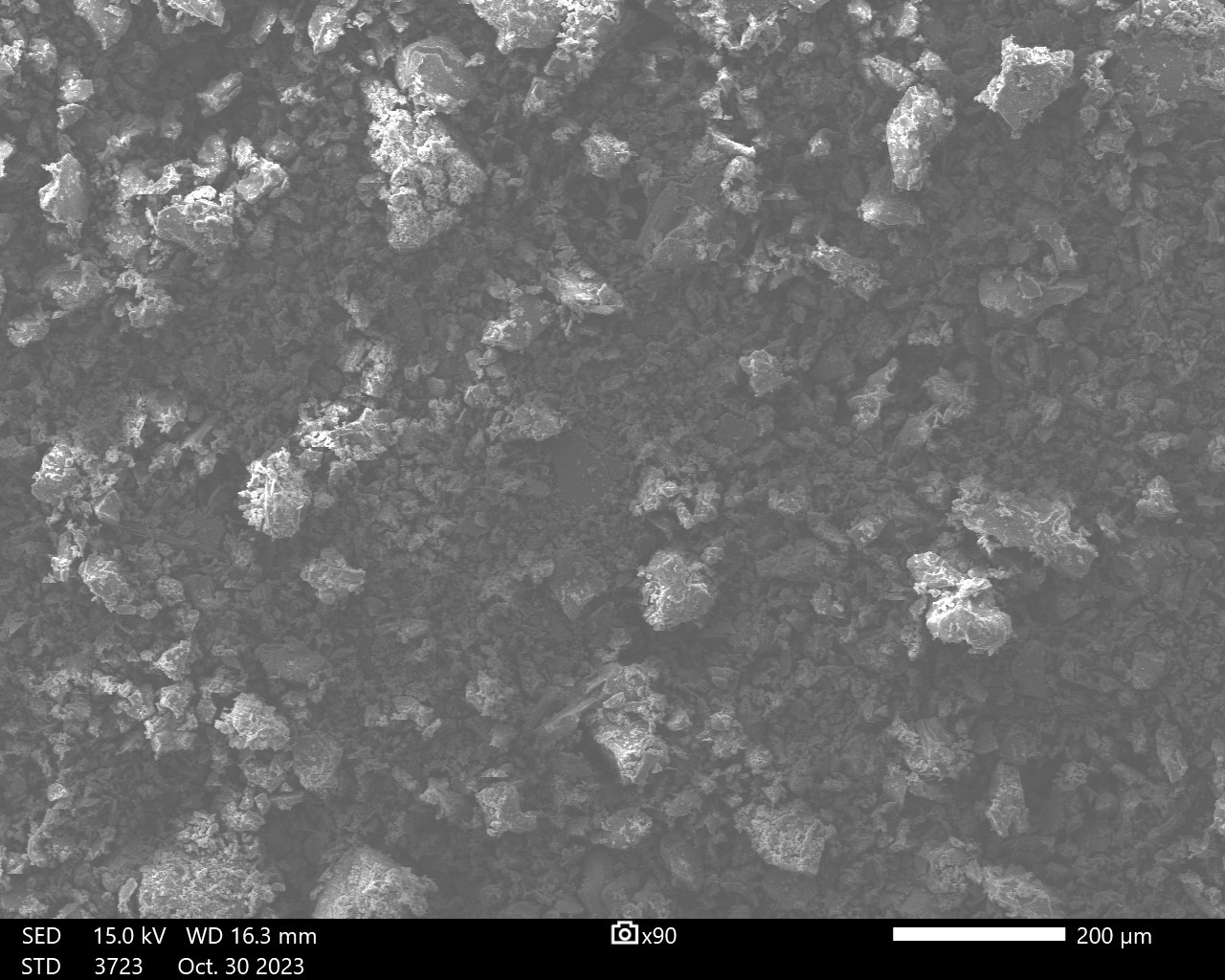

Supplement: S3 Fig — (JPG) [file pone.0314941.s003.jpg]

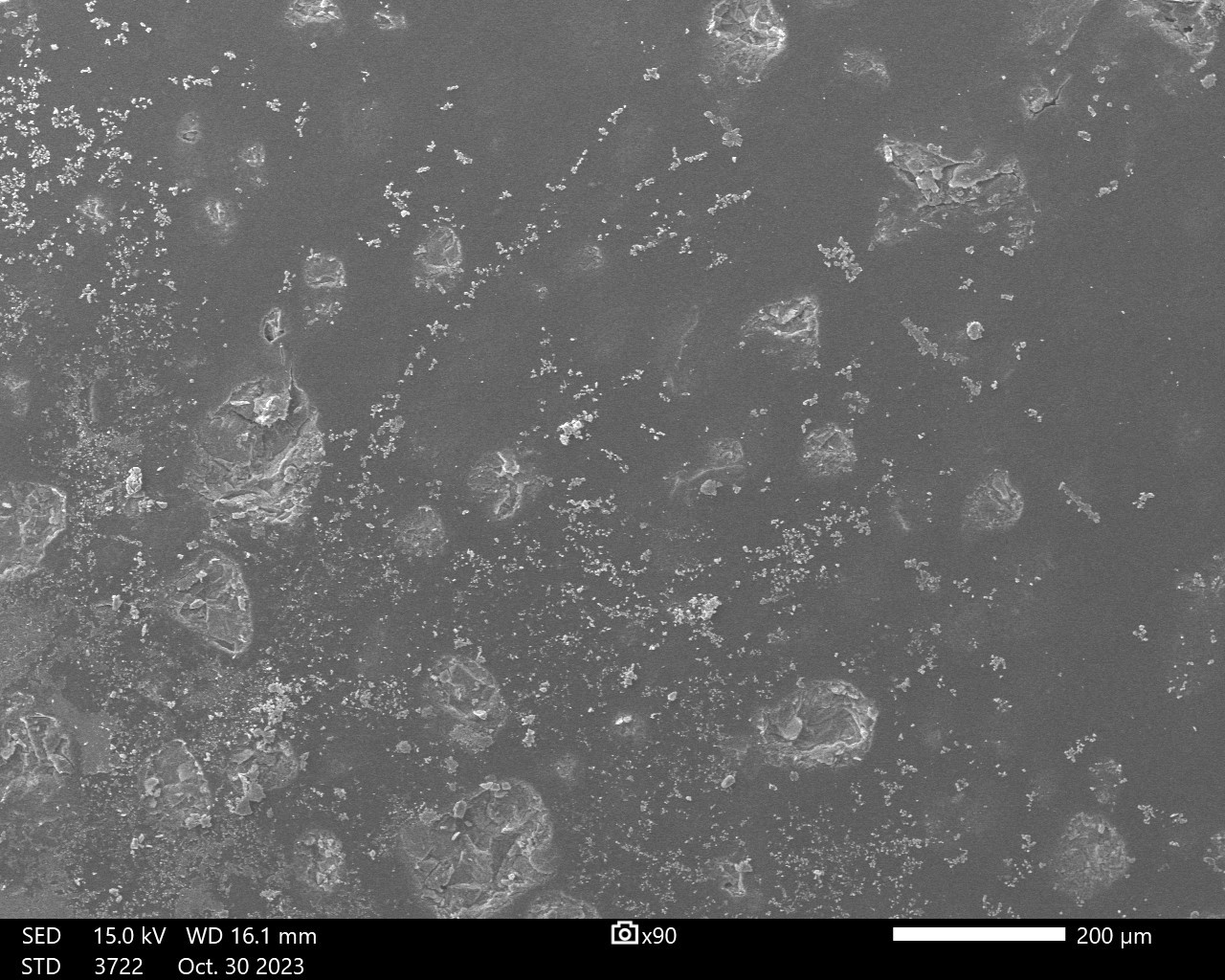

Supplement: S4 Fig — (JPG) [file pone.0314941.s004.jpg]

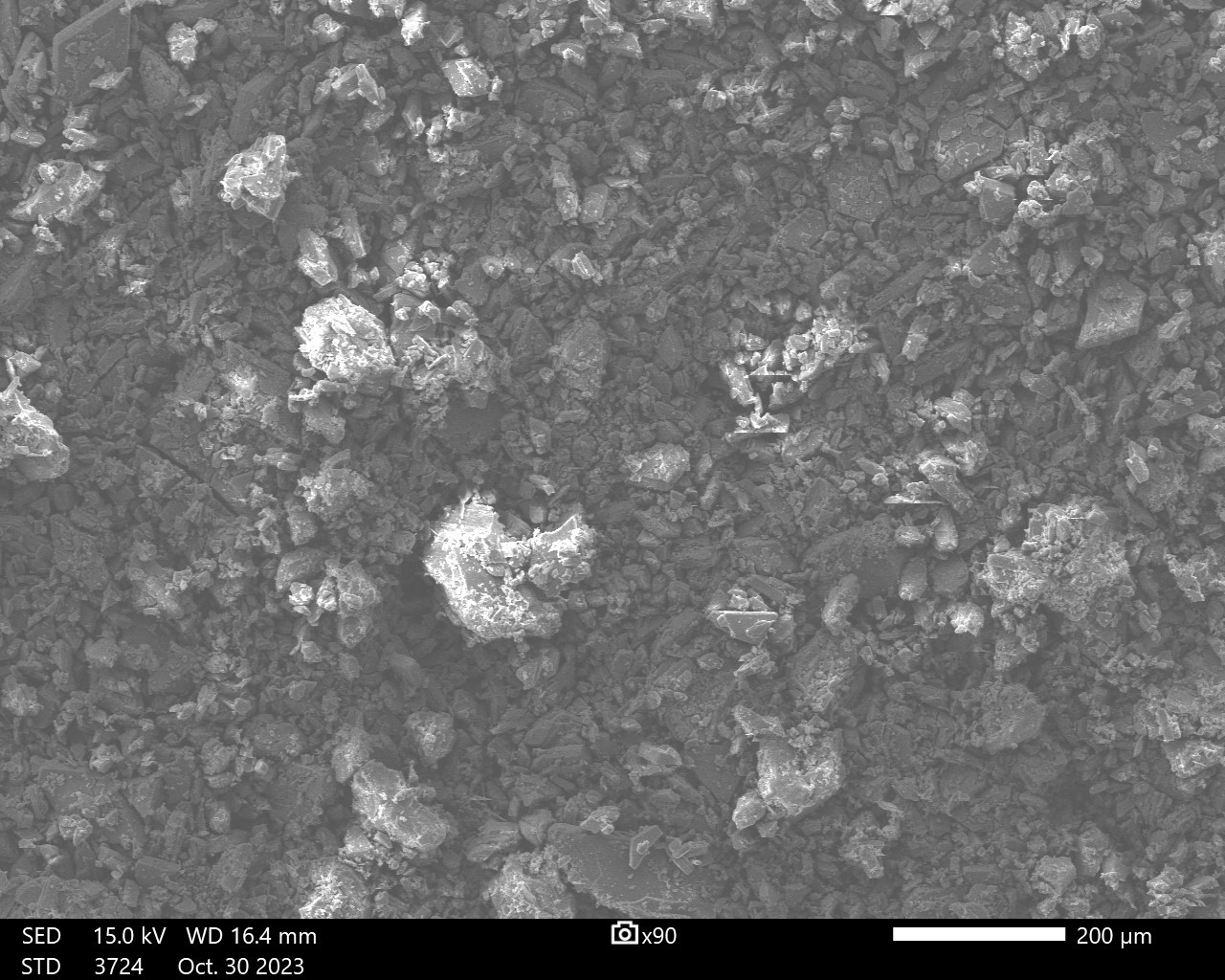

Supplement: S5 Fig — (JPG) [file pone.0314941.s005.jpg]
