# Supplementary material for: Novel methotrexate long-acting system using ambroxol coating and hydroxypropyl methylcellulose encapsulation for preferential and enhanced lung cancer efficiency
Source: PLoS One. 2025 Jan 16;20(1):e0314941. doi: 10.1371/journal.pone.0314941 (PMC11737749; doi:10.1371/journal.pone.0314941)
Supplement: S1 Code — (DOCX) [file pone.0314941.s010.docx]

Code used in the supplementary files

NT-1 =MTX

NT-2= ABL

NT-3= MTX-ABL solid dispersions using (1-1), (1-2), (1-3) and (1-4) ratios, respectively.

NT-4= HPMC-ABL-MTX gel

NT-5= HPMC-ABL blank gel

NT-6= HPMC
