# Supplementary material for: Novel methotrexate long-acting system using ambroxol coating and hydroxypropyl methylcellulose encapsulation for preferential and enhanced lung cancer efficiency
Source: PLoS One. 2025 Jan 16;20(1):e0314941. doi: 10.1371/journal.pone.0314941 (PMC11737749; doi:10.1371/journal.pone.0314941)
Supplement: Attachment — Submitted filename: Response to Reviewers.docx [file pone.0314941.s011.docx]

**Pointwise Response to the Reviewers (Answer in RED):**

**ACADEMIC EDITOR:** 

Please include the following items when submitting your revised manuscript:

- A rebuttal letter that responds to each point raised by the academic editor and reviewer(s). You should upload this letter as a separate file labeled 'Response to Reviewers'.

Answer: Thank you. It was uploaded.

- A marked-up copy of your manuscript that highlights changes made to the original version. You should upload this as a separate file labeled 'Revised Manuscript with Track Changes'.

Answer: Thank you. It was uploaded.

- An unmarked version of your revised paper without tracked changes. You should upload this as a separate file labeled 'Manuscript'.

Answer: Thank you. It was uploaded.

- If you would like to make changes to your financial disclosure, please include your updated statement in your cover letter. Guidelines for resubmitting your figure files are available below the reviewer comments at the end of this letter.

Answer: Thank you. It was included in the cover letter.

- When submitting your revision, we need you to address these additional requirements.

1. Please ensure that your manuscript meets PLOS ONE's style requirements, including those for file naming. The PLOS ONE style templates can be found at

[https://journals.plos.org/plosone/s/file?id=wjVg/PLOSOne_formatting_sample_main_body.pdf](https://c05y1x9s.r.us-east-2.awstrack.me/L0/https:%2F%2Fjournals.plos.org%2Fplosone%2Fs%2Ffile%3Fid=wjVg%2FPLOSOne_formatting_sample_main_body.pdf/1/010f0191bf3b7901-dfd6307d-9267-4aa1-9269-b9e35cffadd8-000000/_DwOGsts-iTL58OQHqFP2UJ5GH4=175) and

[https://journals.plos.org/plosone/s/file?id=ba62/PLOSOne_formatting_sample_title_authors_affiliations.pdf](https://c05y1x9s.r.us-east-2.awstrack.me/L0/https:%2F%2Fjournals.plos.org%2Fplosone%2Fs%2Ffile%3Fid=ba62%2FPLOSOne_formatting_sample_title_authors_affiliations.pdf/1/010f0191bf3b7901-dfd6307d-9267-4aa1-9269-b9e35cffadd8-000000/pcaq8mqzAtw7Hhn_2eye_eyhGMY=175)

Answer: Thank you. It was reflected in the manuscript.

2. Thank you for stating the following financial disclosure:

"Princess Nourah bint Abdulrahman University Researchers Supporting Project number (PNURSP2024R89)"

Please state what role the funders took in the study.  If the funders had no role, please state: "The funders had no role in study design, data collection and analysis, decision to publish, or preparation of the manuscript."

If this statement is not correct you must amend it as needed.

Please include this amended Role of Funder statement in your cover letter; we will change the online submission form on your behalf.

Answer: Thank you. It was included in the cover letter and amended as the following.

“The authors extend their appreciation for the financial support of Princess Nourah bint Abdulrahman University Researchers Supporting Project number (PNURSP2024R89), Princess Nourah bint Abdulrahman University, Riyadh, Saudi Arabia”

3. Your ethics statement should only appear in the Methods section of your manuscript. If your ethics statement is written in any section besides the Methods, please move it to the Methods section and delete it from any other section. Please ensure that your ethics statement is included in your manuscript, as the ethics statement entered into the online submission form will not be published alongside your manuscript.

Answer: Thank you. It was added to the “Materials” section.

4. When completing the data availability statement of the submission form, you indicated that you would make your data available on acceptance. We strongly recommend all authors decide on a data sharing plan before acceptance, as the process can be lengthy and hold up publication timelines. Please note that, though access restrictions are acceptable now, your entire data will need to be made freely accessible if your manuscript is accepted for publication. This policy applies to all data except where public deposition would breach compliance with the protocol approved by your research ethics board. If you are unable to adhere to our open data policy, please kindly revise your statement to explain your reasoning and we will seek the editor's input on an exemption. Please be assured that, once you have provided your new statement, the assessment of your exemption will not hold up the peer review process.

Answer: Thank you. It was included in amended as the follows.

“All data are in the manuscript and/or supporting information files”.

5. We note that the original protocol that you have uploaded as a Supporting Information file contains an institutional logo. As this logo is likely copyrighted, we ask that you please remove it from this file and upload an updated version upon resubmission.

Answer: Thank you. It was uploaded after checking with the IRB issuing institution that it is in the most updated form.

6. Please include captions for your Supporting Information files at the end of your manuscript, and update any in-text citations to match accordingly. Please see our Supporting Information guidelines for more information: [http://journals.plos.org/plosone/s/supporting-information](http://c05y1x9s.r.us-east-2.awstrack.me/L0/http:%2F%2Fjournals.plos.org%2Fplosone%2Fs%2Fsupporting-information/1/010f0191bf3b7901-dfd6307d-9267-4aa1-9269-b9e35cffadd8-000000/5EuV5_eChzpp3e-9qwT9xqzcDqM=175).

Answer: Thank you. It was added as the following at the end of the manuscript.

**“Supporting Information files**

- The institutional review board at Princess Nourah bint Abdulrahman University, Saudi Arabia under the number HAP-01-R-059 was attached.
- The code system included in the experimnets.
- Raw results of calibration curve, Assay, FT-IR, SEM, PXRD, release, MTT and ELISA readings.”

Reviewers' comments:

Reviewer's Responses to Questions

**Comments to the Author**

1. Is the manuscript technically sound, and do the data support the conclusions?

The manuscript must describe a technically sound piece of scientific research with data that supports the conclusions. Experiments must have been conducted rigorously, with appropriate controls, replication, and sample sizes. The conclusions must be drawn appropriately based on the data presented.

Reviewer #1: Yes

Answer: Thank you.

2. Has the statistical analysis been performed appropriately and rigorously?

Reviewer #1: Yes

Answer: Thank you.

3. Have the authors made all data underlying the findings in their manuscript fully available?

The [PLOS Data policy](http://c05y1x9s.r.us-east-2.awstrack.me/L0/http:%2F%2Fwww.plosone.org%2Fstatic%2Fpolicies.action%23sharing/1/010f0191bf3b7901-dfd6307d-9267-4aa1-9269-b9e35cffadd8-000000/z7A0Q6-bjG5XudJ4qScVhI58IYM=175) requires authors to make all data underlying the findings described in their manuscript fully available without restriction, with rare exception (please refer to the Data Availability Statement in the manuscript PDF file). The data should be provided as part of the manuscript or its supporting information, or deposited to a public repository. For example, in addition to summary statistics, the data points behind means, medians and variance measures should be available. If there are restrictions on publicly sharing data—e.g. participant privacy or use of data from a third party—those must be specified.

Reviewer #1: Yes

Answer: Thank you.

4. Is the manuscript presented in an intelligible fashion and written in standard English?

PLOS ONE does not copyedit accepted manuscripts, so the language in submitted articles must be clear, correct, and unambiguous. Any typographical or grammatical errors should be corrected at revision, so please note any specific errors here.

Reviewer #1: No

Answer: The authors would like to extend their appreciation to the reviewer. A professional language editing service was requested and applied in the revised version of the manuscript.

5. Review Comments to the Author

Please use the space provided to explain your answers to the questions above. You may also include additional comments for the author, including concerns about dual publication, research ethics, or publication ethics. (Please upload your review as an attachment if it exceeds 20,000 characters)

Reviewer #1: To identify potential negative points regarding the research study described in the manuscript, consider the following aspects:

1. The study mentions that methotrexate (MTX) has poor selectivity as an immunosuppressant, which is a fundamental challenge.

Answer: The authors would like to appraise their appreciation for the reviewer’s point. The developed system was designed to elicit the selectivity profile as it was proven on the cellular level after 24 hours of treatment to increase the expression level of Folate Receptor-ɑ (FR-ɑ). The MTX lung cancer uptake of the MTX-ABL (1: 4) solid dispersion was superior to the other groups which could indicate the enhanced lung cancer uptake. The FR-ɑ is highly expressed in the A549 lung cancer cells and has been involved in the MTX mechanism of action. However, a long-term *in vivo* absorption study will be recommended to solidify the claim (ADDED to the Conclusions).

2. The study does not provide long-term resistance data, which could be a limitation.

Answer: The authors would like to confirm that the long-term *in vivo* absorption study will be recommended to solidify the claim and identify the resistance profile developed (ADDED to the Conclusions).

3. Ambroxol, despite its anticancer properties, is traditionally used as a mucolytic agent, and its long-term effects when repurposed for cancer therapy are not fully understood.

Answer: ABL was tested by the first author in previous articles on lung cancer repurposing (Md et al., 2021). We can affirm that the authors will take the reviewer's insight into the next investigation on the molecular level and signalling to identify how ABL can elicit the BAX expression in cancer cells. Interestingly, ABL was proven to boost the phagocytosis of lung cancer dead cells after the chemotherapeutic treatment in an *in vivo* trial as published before (Zhang et al., 2017). This could encourage that the data in the *in vivo* trial is worthy of investigation (ADDED to the Conclusions).

4. The study is primarily focused on in vitro tests only using A549 lung cancer cells!

Answer: The authors would like to confirm that the long-term *in vivo* absorption study will be recommended to solidify the claim and identify the resistance profile developed (ADDED to the Conclusions).

5. The manuscript needs extensive revision for language and grammar. Some editing for the English language is required throughout the manuscript due to too many mistakes.

Answer: The authors would like to extend their appreciation to the reviewer. A professional language editing service was requested and applied in the revised version of the manuscript (ADDED to the Conclusions).

6. The quality of some of the figures should be improved.

Answer:

Thank you. The authors have enhanced the figure quality to support the claim given. Kindly note that the submission system file decreases the Figures' quality in the PDF. Authors are kindly advised to download the figures separately (ADDED to the Conclusions).

**References**

Md, S., Abdullah, S. T., Alhakamy, N. A., Bani-Jaber, A., Radhakrishnan, A. K., Karim, S., . . . Rizg, W. Y. (2021). Ambroxol Hydrochloride Loaded Gastro-Retentive Nanosuspension Gels Potentiate Anticancer Activity in Lung Cancer (A549) Cells. *Gels, 7*(4). doi:10.3390/gels7040243

Zhang, X., Chen, Q., Chen, M., Ren, X., Wang, X., Qian, J., . . . Sha, X. (2017). Ambroxol enhances anti-cancer effect of microtubule-stabilizing drug to lung carcinoma through blocking autophagic flux in lysosome-dependent way. *Am J Cancer Res, 7*(12), 2406-2421.
